# Fluoride anodic films on stainless-steel fomites to reduce transmission infections

**DOI:** 10.1128/aem.01892-23

**Published:** 2024-01-30

**Authors:** Ana Conde, Daniel Voces, Marina Medel-Plaza, Celia Perales, Ana Isabel de Ávila, John Jairo Aguilera-Correa, Juan Jose de Damborenea, Jaime Esteban, Esteban Domingo, Maria Angeles Arenas

**Affiliations:** 1 Centro Nacional de Investigaciones Metalúrgicas, CENIM-CSIC, Madrid, Spain; 2 CIBERINFEC, CENTRO DE INVESTIGACIÓN BIOMEDICA EN RED Enfermedades Infecciosas, Madrid, Spain; 3 IIS-Fundación Jiménez Díaz, IIS-FJD, Madrid, Spain; 4 Centro Nacional de Biotecnología, CNB-CSIC, Madrid, Spain; 5 CIBEREHD, CENTRO DE INVESTIGACIÓN BIOMEDICA EN RED Enfermedades Hepáticas y Digestivas, Madrid, Spain; 6 Centro de Biología Molecular Severo Ochoa (CBMSO) (CSIC-UAM), Madrid, Spain; 7 Pathogénie mycobactérienne et nouvelles cibles thérapeutiques, Institut de Recherche en Infectiologie de Montpellier, Montpellier, France; Centers for Disease Control and Prevention, Atlanta, Georgia, USA

**Keywords:** anodic films, fluoride, antimicrobial, SS304L

## Abstract

The growing concern arising from viruses with pandemic potential and multi-resistant bacteria responsible for hospital-acquired infections and outbreaks of food poisoning has led to an increased awareness of indirect contact transmission. This has resulted in a renewed interest to confer antimicrobial properties to commonly used metallic materials. The present work provides a full characterization of optimized fluoride anodic films grown in stainless steel 304L as well as their antimicrobial properties. Antibacterial tests show that the anodic film, composed mainly of chromium and iron fluorides, reduces the count and the percentage of the area covered by 50% and 87.7% for *Pseudomonas aeruginosa* and *Stenotrophomonas maltophilia*, respectively. Virologic tests show that the same treatment reduces the infectivity of the coronavirus HCoV-229E-GFP, in comparison with the non-anodized stainless steel 304L.

**IMPORTANCE:**

The importance of environmental surfaces as a source of infection is a topic of particular interest today, as many microorganisms can survive on these surfaces and infect humans through direct contact. Modification of these surfaces by anodizing has been shown to be useful for some alloys of medical interest. This work evaluates the effect of anodizing on stainless steel, a metal widely used in a variety of applications. According to the study, the fluoride anodic layers reduce the colonization of the surfaces by both bacteria and viruses, thus reducing the risk of acquiring infections from these sources.

## INTRODUCTION

The survival of pathogenic microorganisms on inanimate surfaces contributes to the persistence and indirect transmission of infectious agents. Metallic materials and, particularly austenitic stainless steels, are part of our daily life due to their good combination of mechanical and corrosion properties, cleanability, and esthetic features. These features render these materials suitable to manufacture items used in architecture or public transport, such as elevators, benches, railings, and other types of handholds that are in contact with many individuals within short time intervals. Such materials are also common in medical settings and the food industry.

Contaminated surfaces are an established route of transmission for important nosocomial pathogens including methicillin-resistant *Staphylococcus aureus*, vancomycin-resistant enterococci, and norovirus, which share the ability to survive for extended periods (1, 2). Enveloped viruses, such as influenza and human coronaviruses including Middle East respiratory syndrome coronavirus (MERS-CoV) and Severe acute respiratory syndrome coronavirus (SARS-CoV), have a limited capacity to survive on dry surfaces (3, 4). For example, the risk of surface transmission of SARS-CoV-2 has been estimated by the Centers for Disease Control and Prevention (CDC) to be less than 1 in 10,000 (5). Despite this low risk, when the environmental conditions are favorable coronaviruses can persist for several days (6).

Cleaning routines using disinfectants have demonstrated a reduction in the rate of transmission of influenza virus and diarrheal disease (7, 8). The use of antimicrobial surfaces can be a complementary strategy to disinfection and cleaning to preserve stainless steel from pathogen contamination. Different approaches have been used to modify surfaces and provide them with antimicrobial properties. They include surface topography, roughness, or nanostructure modifications to alter their hydrophilic/phobic properties and to change their surface composition, by incorporating either organic or inorganic compounds (9), copper, and silver (10–12). These treatments have proven effective in inhibiting viral/bacterial adhesion or even providing materials with viricidal and bactericidal activity (13).

Several studies have documented that surface topography and roughness of stainless steel in connection with bacterial size are key factors in promoting bacterial adhesion and retention, as well as reducing the cleanability of the surface. Similarly, wettability and surface energy are also relevant properties in the adhesion process. Surface free energy (or surface energy) is the excess energy the surface has compared to the bulk material. This results from an imbalance of forces at the surface compared to the bulk of the material, where molecules are surrounded by similar molecules and pulled equally in all directions, resulting in a zero-net force on each molecule. In contrast, at the surface (air/solid interface), the material only has similar adjacent molecules on one side, while on the other side, there is very little interaction with the molecules in the air, resulting in excess energy at the solid interface. Quantifications of surface energy require at least two probe liquids. However, it can be roughly predicted by measuring the water contact angle. If the contact angle is <90°, the water spreads on the solid surface, the liquid wets the surface, and the surface free energy is high (the surface is hydrophilic). Conversely, if the contact angle is >90°, the water does not wet the surface and the energy is low (the surface is hydrophobic) (14). In general, hydrophobic surfaces appear to be more susceptible to bacterial adhesion than hydrophilic ones. Surface energy, which also depends on the condition layer (environment) and surface structure, is an important factor influencing bacterial adhesion (15).

*Pseudomonas aeruginosa* and *Stenotrophomonas maltophilia* are able to form biofilm on both hospital and household surfaces, causing mostly healthcare-associated infections but also community-associated infections. *P. aeruginosa* is considered the most dangerous microorganism and it is listed as a priority pathogen for Research and Development of new antibiotics by the World Health Organization (16, 17). Moreover, the ability to form biofilm is a recognized trait of *S. maltophilia*, but its clinical relevance is still unclear (18). However, its extraordinary ability to adhere to inanimate surfaces and its multi-resistant nature make it a critical pathogen in the healthcare environment (18, 19). These two species are examples of environmental bacteria that can be true pathogens, especially among patients in the intensive care units, immunosuppressed hosts, or patients with other conditions that make them susceptible to infection.

This work aims at providing additional properties to stainless-steel surfaces to inhibit pathogen adherence and to reduce their persistence, by incorporating fluorine, F, in the surface by means of an anodizing process. The antimicrobial properties of fluorine are widely used in dental health (20). Fluoride can affect bacterial metabolism as an enzyme inhibitor. Metal-fluoride complexes are also responsible for fluoride inhibition of proton-translocating F-ATPases, thus reducing the acid tolerance of the bacteria; they are thought to mimic phosphate to form complexes with Adenosine diphosphate (ADP) at the catalytic sites of the enzymes (21, 22).

However, the literature regarding the incorporation of F in metallic surfaces is scarce, and it is mainly focused on titanium alloys for biomedical use. Some works about the incorporation of F in Ti alloys by means of ion implantation and anodizing process have shown different antimicrobial efficiency (23–25). Nanostructured fluoride anodic films in titanium alloys reduce bacterial adherence by 50%. In these studies, the antibacterial properties were tested *in vitro* against *S. aureus*, *Staphylococcus epidermidis*, and *P. aeruginosa* using both collection and clinical strains (25–27).

Anodizing is a well-established process to provide corrosion resistance on valve metals (Al, Ti, and Mg). Recently, the growth of anodic films in iron-base alloys in fluoride-containing solutions has attracted much interest due to their potential applications in solar cells, photocatalysis and hydrogen production (28), nanohole arrays for fabricating functional devices (29), or even to tailor surface hydrophilicity for biomedical applications (30).

In most of these applications, the anodic layers are subjected to a thermal treatment to remove fluorine to gain stability of the layer. Preliminary work developed by the authors has demonstrated that it is possible to grow stable fluoride anodic layers in AISI 304 stainless steel by anodizing in organic baths with fluoride additives (31).

The present paper assesses the antimicrobial properties of the fluoride anodic films grown on 304L stainless steel using two laboratory strains of *P. aeruginosa* and *S. maltophilia*, and the coronavirus HCoV-229E-GFP. Antimicrobial tests show a reduction in the surface area covered by both bacterial strains and a lower infectivity of the coronavirus HCoV-229E-GFP compared to non-anodized 304L.

## MATERIALS AND METHODS

### Surface modification

Disc samples of 3 mm of thickness of stainless steel 304L (18.29 wt.% Cr, 8.04 wt.% Ni, 1.43 wt.% Mn, 0.31 wt.% Mo, 0.42 wt.% Si, 0.023 wt.% C, bal. Fe) were prepared from a commercial cold-drawn bar of 15 mm diameter. The surface of the samples was ground using successive SiC sandpaper from 200 to 3,000 grit and, subsequently, polished with a diamond paste of 3 μm. Afterward, the samples were rinsed and cleaned with distilled water and ethanol, and then dried in an air stream.

The anodizing process was carried out in a two-electrode cell using a platinum foil coupon of 2.25 cm² as cathode and the 304L discs as anode. Just one side of the disc of 1.77 cm² was anodized in an ethylene glycol (EG) electrolyte containing 0.1 M NH_4_F and 0.1 M H_2_O in static conditions. The anodizing process was accomplished at a constant voltage and a temperature of 5 ± 1°C. The voltage was applied in ramp mode at a rate of 1 V s^−1^ up to 50 V and then, this voltage was kept constant for 15 min. Samples were immersed in a saturated CaCO_3_ solution to remove fluorides, subsequently cleaned with distilled water, rinsed with ethanol, and then dried in an air stream.

### Surface characterization

Before and after anodizing the roughness of the surface was characterized by a Sensofar plμ2300 optical imaging profiler using an objective 20× EPI magnification. Surface roughness measurement was performed in an area of 557 × 398 µm². Data processing was done according to ISO 25178 standard using a Gaussian L filter (*λ*_*c*_= 80 × 80 µm). Three distinct regions were analyzed on each surface condition. Normality of each series of data was checked with the Shapiro-Wilk test using Origin software with a significance level of *P* < 0.05. Statistical significance was evaluated using analysis of variance by Levene’s test. The average surface roughness parameter, *Sa,* is presented as the mean value and the standard deviation (*X* ± standard deviation).

X-ray diffraction analysis has been carried out in a Bruker D8 Advance X-ray diffractometer with a Co anode and operating in grazing mode at a fixed angle of 2°.

Nanostructure of the anodic oxide layer was first analyzed by a field emission gun scanning electron microscope (FEG-SEM) Hitachi S 4800 J equipped with energy-dispersive X-ray spectroscopy (EDX). The stoichiometric composition and thickness of the oxide films were further determined by Rutherford Backscattering Spectrometry (RBS), using He^+^ ions with an energy of 3.035 MeV (resonant energy for ^16^O(α, α_0_)^16^O reaction), produced by the van de Graff accelerator at The Centre of Micro Analysis of Materials, UAM, Madrid. The incident ion beam, with a diameter of 1 mm, was normal to the specimen surface with 10 μC dose scattered ions detected by a fixed detector at 170°. Data were analyzed using the SIMNRA program.

### Characterization of the antimicrobial properties

#### Bacterial adherence

Two collection strains, known as environmental bacteria capable of developing biofilm and causing infections, such as *P. aeruginosa* ATCC 27853 (26, 32, 33) and *S. maltophilia* ATCC 13637 (34–36) were used. All the strains were stored at −80°C until the experiments were started.

The bacterial adhesion experiments on the non-anodized and anodized 304L steel samples were performed following a modification of the methodology previously described by Aguilera-Correa et al. (37). Each sample was washed and vortexed for 15 s at 300 rpm in pure distilled water (B. Braun, Germany) before this experiment was performed. Each strain was grown in tryptic soy broth (bioMérieux, Marcy-l’Étoile, France) at 37°C for 24 h. After culture, bacteria were harvested at 3,500 rpm for 10 min. The supernatant was discarded, and the pellet was washed three times with sterile 0.9% NaCl saline solution (SS) (B. Braun). Bacteria were then suspended and diluted in SS, reaching 10^8^ CFU mL^−1^ bacterial solution, and 5 mL of this solution was statically incubated on 304L steel samples in a sterile nontreated six-well plate (Thermo Fisher Scientific, MA, USA) at 22°C for 90 min (25, 38, 39). After incubation, samples were washed three times with SS to remove non-adhered bacteria, as described in the literature (38). Metallic samples were then stained with a Live/Dead Bac Light bacterial viability kit (Thermo Fisher Scientific, MA, USA) and rinsed with sterile water (40). About 10 photographs of different fields (400× magnifications) were taken with a DM 2000 fluorescence microscope (Leica Microsystems, Wetzlar, Germany) for each sample. All images were taken using the same microscopy conditions (380–463.1-ms exposure time, 5.5× optical gain, 1.50 saturation level, and gamma of 10.00). The percentage of the total surface covered with adhered bacteria as well as the percentages of dead and live bacteria were obtained by using ImageJ software (National Institutes of Health, Bethesda, MD, USA) as previously described (37). The experiments were performed in triplicate for each strain.

Finally, after 90 min of incubation, bacterial solution was used to estimate the number of CFU mL^−1^ of planktonic bacteria exposed to each material using the drop plate method (41) on MacConkey agar (bioMérieux, Marcy-l’Étoile, France).

The statistical analysis was performed by using GraphPad Prism 8.0.2 software (Dotmatics, San Diego, CA, USA), and data were analyzed by the nonparametric unilateral Wilcoxon test with a level of statistical significance of *P* < 0.05. The values are cited and represented as medians and interquartile ranges.

#### Cells and viruses

Huh-7 cells were grown in Dulbecco’s modified Eagle’s medium (DMEM, Merck) supplemented with 1 mM sodium pyruvate (Merck), 1% non-essential amino acids (Merck), 4 mM L-glutamine (Merck), 50 µg mL^−1^ gentamicin (Panreac), 100 U mL^−1^ penicillin, 100 µg mL^−1^ streptomycin, 0.2 µg mL^−1^ antifungal (Sigma), and 10% fetal bovine serum (FBS) (Sigma). Cells were cultured at 37°C and 5% CO_2_, and they were periodically thawed from a large frozen stock and passaged a maximum of 30 times at a split ratio of 1:4 to 1:5.

The virus used in the experiments was HCoV-229E-GFP. To prepare a virus stock, 3 × 10^7^ Huh-7 cells were infected with the virus at a multiplicity of infection of 0.01 plaque-forming units (PFUs)/cell in DMEM supplemented with 10% FBS, and the infection was allowed to proceed for 96 h at 33°C. The titer of the viral stock was 3.2 × 10^7^ PFUs mL^−1^. To control for the absence of contamination, the supernatants of mock-infected cells, which were maintained in parallel with the infected cultures, were titrated; no infectivity in the mock-infected cultures was detected in any of the experiments.

Four different viral loads (3 × 10^6^, 3 × 10^5^, 3 × 10^4^, and 3 × 10^3^ PFU) were incubated on anodized and non-anodized samples with a final volume of 100 µL on a surface sample of 1.77 cm². The samples were previously sterilized by subjecting them to 160°C for 2.5 h. The contact time of the virus on the surface was approximately 1 h. The virus was re-suspended in 1 mL DMEM using mild vortexing. To control for the absence of contamination, samples coated with DMEM in the absence of virus were maintained in parallel.

Virus titrations were performed in Huh-7 cells following standard procedures. For titration of infectious HCoV-229E-GFP, viruses eluted from the anodized and non-anodized samples were serially diluted and applied to 1 × 10^6^ Huh-7 cells. After 2 h adsorption with gentle stirring every 15 min, the inoculum was removed, and medium containing DMEM 2×, agar 1.4% (Gibco), 2% FBS, and 1% DEAE-Dextran (Sigma) was added to the plates. After 96 h, cells were fixed with 2% formaldehyde (Panreac) for 20 min, and then stained with 2% crystal violet (Merck) in formaldehyde, for plaque counting and titer calculation.

## RESULTS AND DISCUSSION

**Fig 1:**
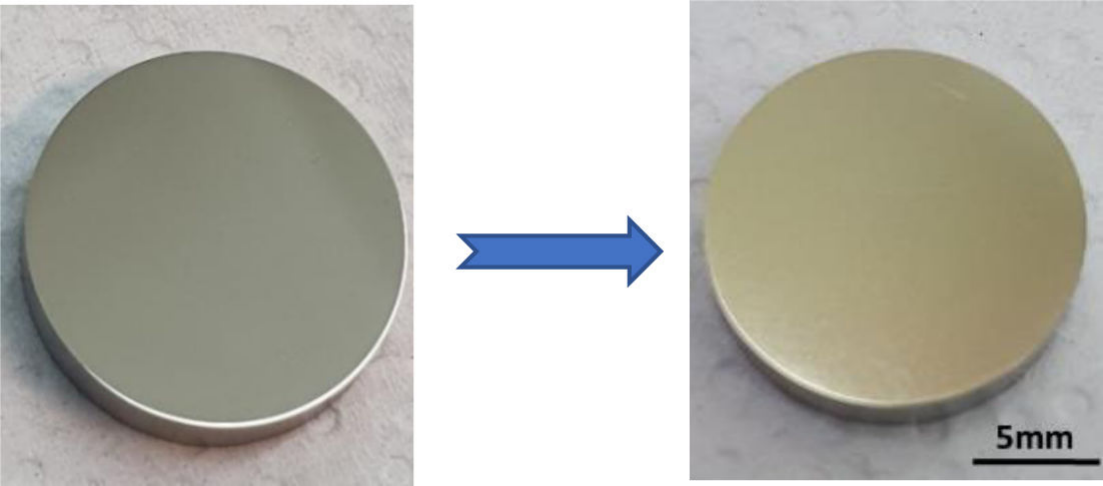
Color change of the 304L stainless-steel surface as a result of the anodizing process.

As a result of the anodizing process of the SS 304L in EG electrolyte containing 0.1 M NH_4_F and 0.1 M H_2_O at 5 ± 1°C, the samples experience a slight color change toward a yellowish appearance (Fig. 1) and an increase in surface roughness from *Sa* 13.9 ± 0.7 nm (for non-anodized 304L stainless-steel sample) to 101 ± 6 nm (for anodized sample).

**Fig 2:**
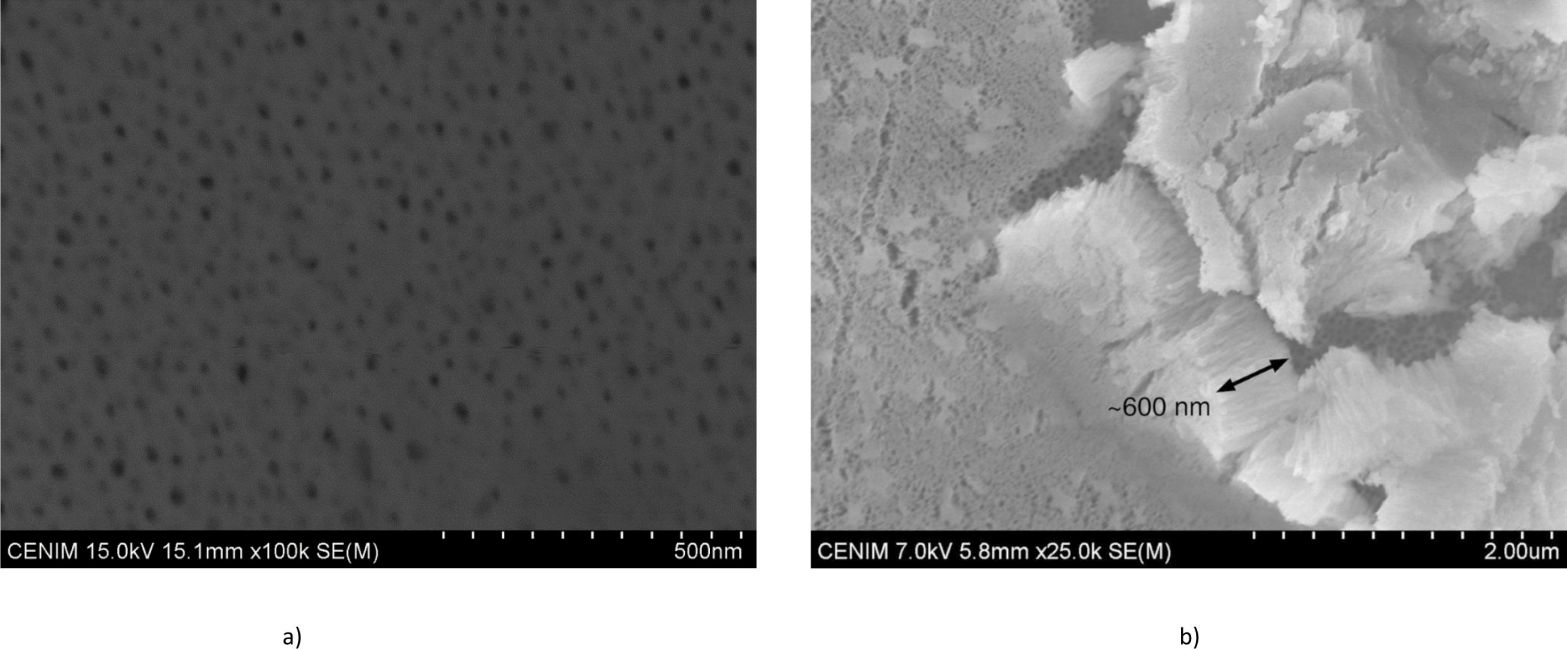
SEM images. (**a**) Plan view of the anodic oxide film. (**b**) Area of the anodic film broken and detached by the incision made with the scalpel.

The SEM analysis reveals that the anodic film has a porous nanostructure (Fig. 2a) with a wide distribution of pore diameters from ~10 nm to ~40 nm. The thickness of the anodic oxide film, measured in an area intentionally scratched by a scalpel to break and detach the film (Fig. 2b), was ~600 nm. The analysis of the anodic film performed by EDX revealed that it is mainly composed of F, Fe, and Cr (~53.99 at.%, 27.71 at.%, and 9.91 at.%, respectively) with minor contents of O, Ni, and Si (5.28 at.%, 2.58 at.%, and 0.53 at.%, respectively). These results suggest that the anodic layer is mainly composed of iron and chromium fluorides and to a lesser extent of their oxides.

**Fig 3:**
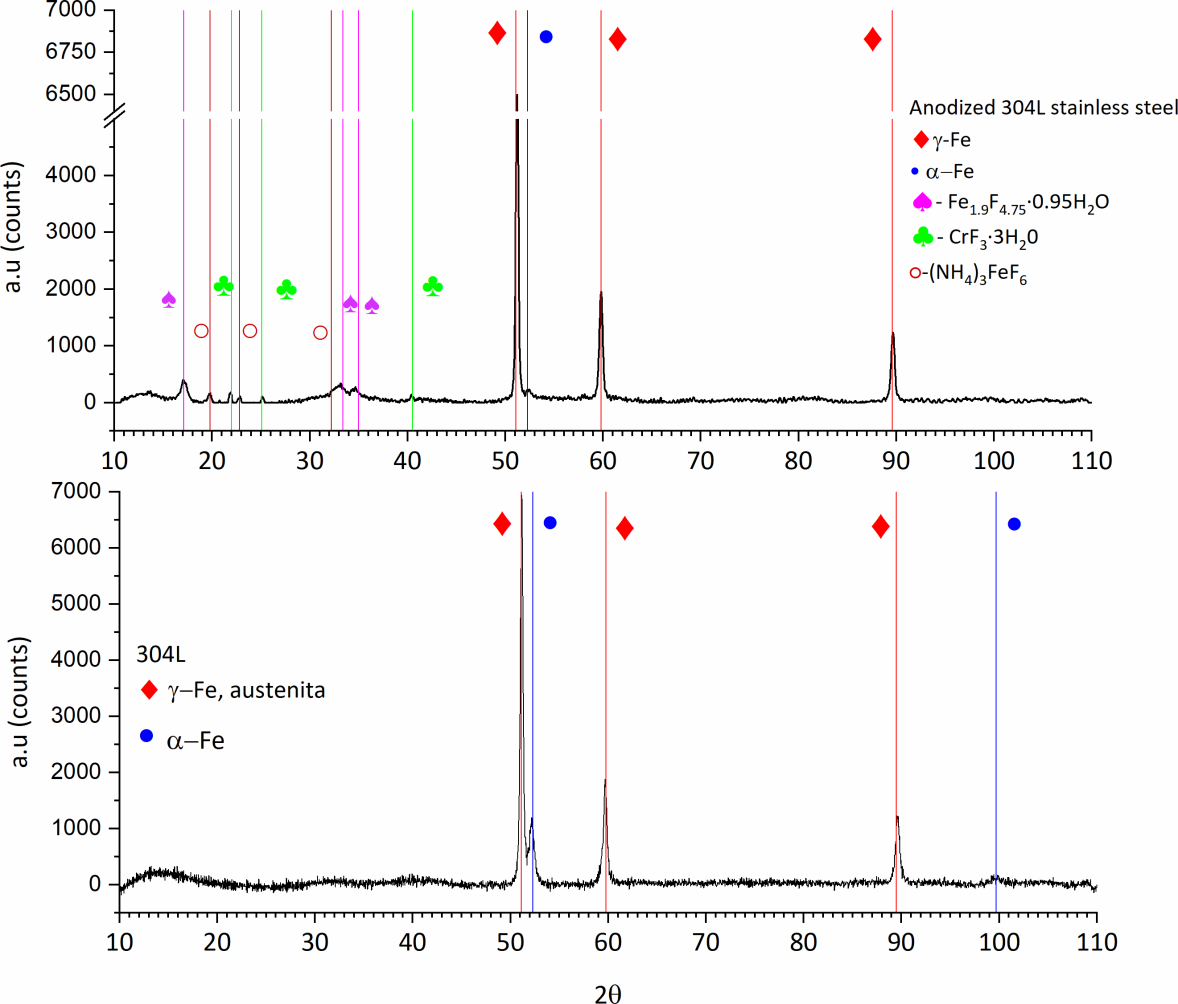
X-ray diffractograms corresponding to anodized and non-anodized 304L stainless steel.

Further analysis by X-ray diffraction in grazing mode confirmed the formation of fluoride compounds in the anodized 304L samples, whereas the non-anodized 304L samples showed strong peaks corresponding to γ-Fe (austenite) and α-Fe (ferrite) (Fig. 3). The XRD studies conducted on anodized samples showed additional peaks corresponding to hydrated chromium fluoride and iron fluoride, as well as iron ammonium fluoride. Klimas et al. (42) have reported similar results for anodic films grown in a glycerol solution containing NH_4_F and low water additions but at higher temperatures and anodizing voltages (60°C and 70 V) than those used in the present work.

**Fig 4:**
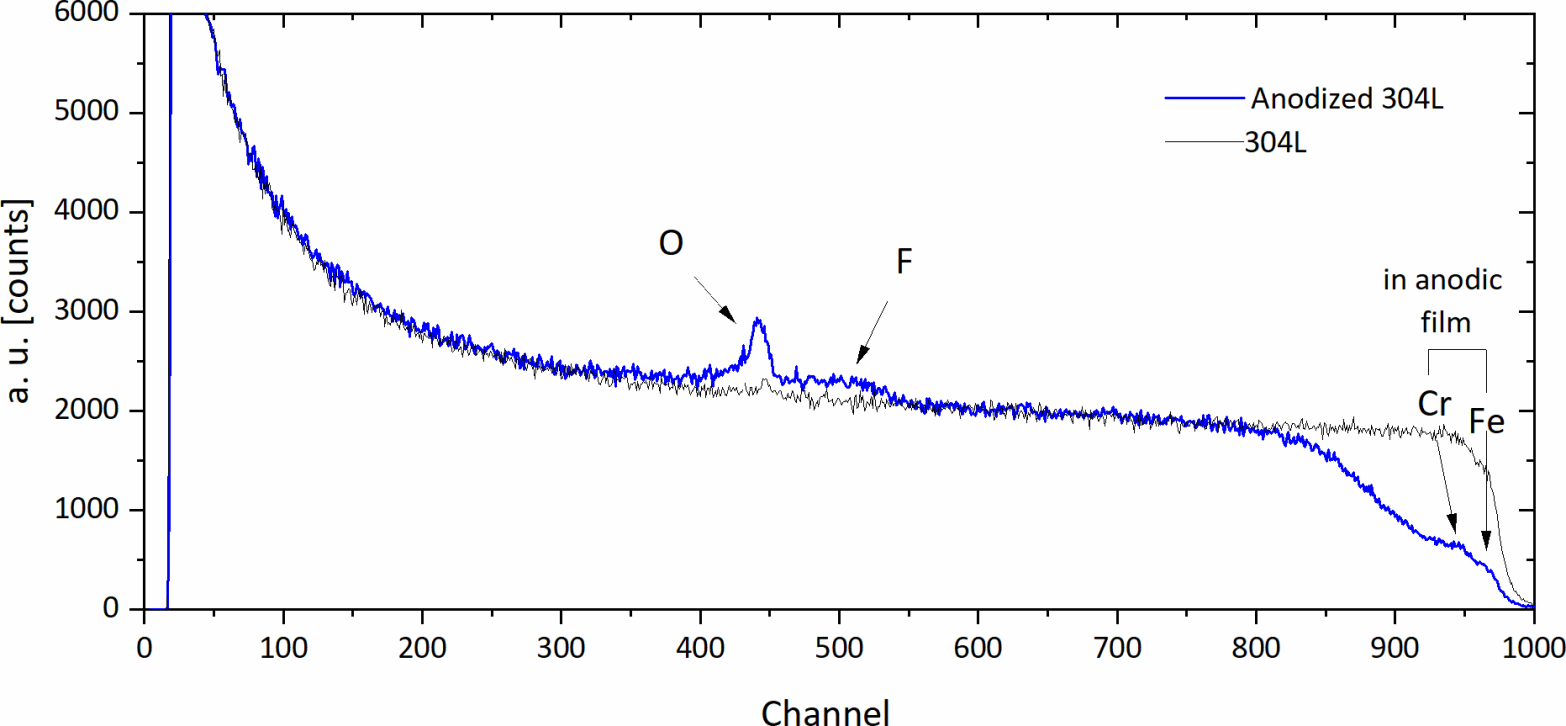
Comparison of RBS spectra of anodized and non-anodized 304L stainless steel.

The composition and thickness of the anodic films were also examined by RBS. Figure 4 compares the RBS spectra corresponding to the anodized and non-anodized 304L stainless-steel samples. The yields from fluorine and oxygen in the anodic film appear separately from the Cr and Fe yields.

The average molecular composition of the anodic layer estimated from the RBS analysis is gathered in Table 1. The anodic film comprised an inner fluoride-enriched layer of ~95.5 nm thickness and an outer layer ~511 nm thick mainly composed of iron fluoride hydroxide, chromium fluoride, and chromium oxide. The total thickness of the anodic layer estimated from the RBS is ~606.5 nm which is also consistent with the value measured at the SEM micrographs.

**TABLE 1:** Average molecular composition from RBS analysis of the anodic film

Layer	Average molecular composition	Thickness (nm)
**Nanoporous anodic film**		
Outer	FeF_1.98_(OH)_0.58_ · 0.011CrF_3_ · 0.011Cr_2_O_3_	511
F-enriched	FeF_0.843_	95.5
Total thickness		606.5

The literature describes the formation of such thick oxygen-free F-enriched layer of FeF_*x*_ at the metal/film interface as a consequence of the field-assisted-dissolution growth mechanism of anodic layer (43) [being FeF_3_ (44) or FeF_2_ (45)] due to the smaller ionic radius and faster migration rate of fluoride than oxygen ions (O^2−^).

The chemical composition of the anodic layer grown in 304L stainless steel notably differs from that grown in pure Fe. Fadillah et al. (45) reported that for pure iron, the formation of an anodic layer composed of Fe_2_O_3_ · FeF_2_ on (100) facet, whereas Fe_3_O_4_ · FeF_2_ formed on a higher index number facet. These authors state that the presence of FeF_3_ in the anodic film is negligible due to its faster chemical dissolution in the electrolyte regarding FeF_2_ due to the difference in their solubility constants. Conversely, in this work, the anodic film fabricated on 304L stainless steel is mainly composed of FeF_*X*_(OH)_*Y*_ with minor contents of CrF_3_ and Cr_2_O_3._

This anodic layer shows a preferential composition in chromium oxides and metallic fluorides than iron oxides. The low oxide content of the anodic film is explained according to the standard thermodynamic values. Indeed, the Gibbs free energy formation of the different chromium and iron oxides and fluorides reveals that Cr_2_O_3_ (−1,058.1 KJ mol^−1^), CrF_3_ (−1,088 KJ mol^−1^), and FeF_3_ (−972 KJ mol^−1^) are thermodynamically favorable compared to Fe_2_O_3_ (−742.2 KJ mol^−1^) and FeF_2_ (−668.6 KJ mol^−1^) (36).

Thus, the different analyses performed to establish the composition of the anodic film (XRD, EDX, and RBS) grown in 304L stainless steel confirm that the anodic film is mainly composed of iron fluoride hydroxide, chromium oxide, and chromium fluoride.

### Antimicrobial properties

Antibacterial properties of the fluoride anodic film grown in 304L stainless steel were tested using collection strains of environmental non-fermentative Gram-negative bacilli such as *P. aeruginosa* and *S. maltophilia*. Both types of bacteria show persistence on dry surfaces for several months. The type of bacteria appears to have some influence on survival times since Gram-negative bacteria show longer persistence times in comparison to Gram-positive (1, 25, 38). Moreover, the influence of the type of material appears unclear since the results are strongly dependent on the experimental conditions; therefore, many of the published results are inconsistent (1).

**Fig 5:**
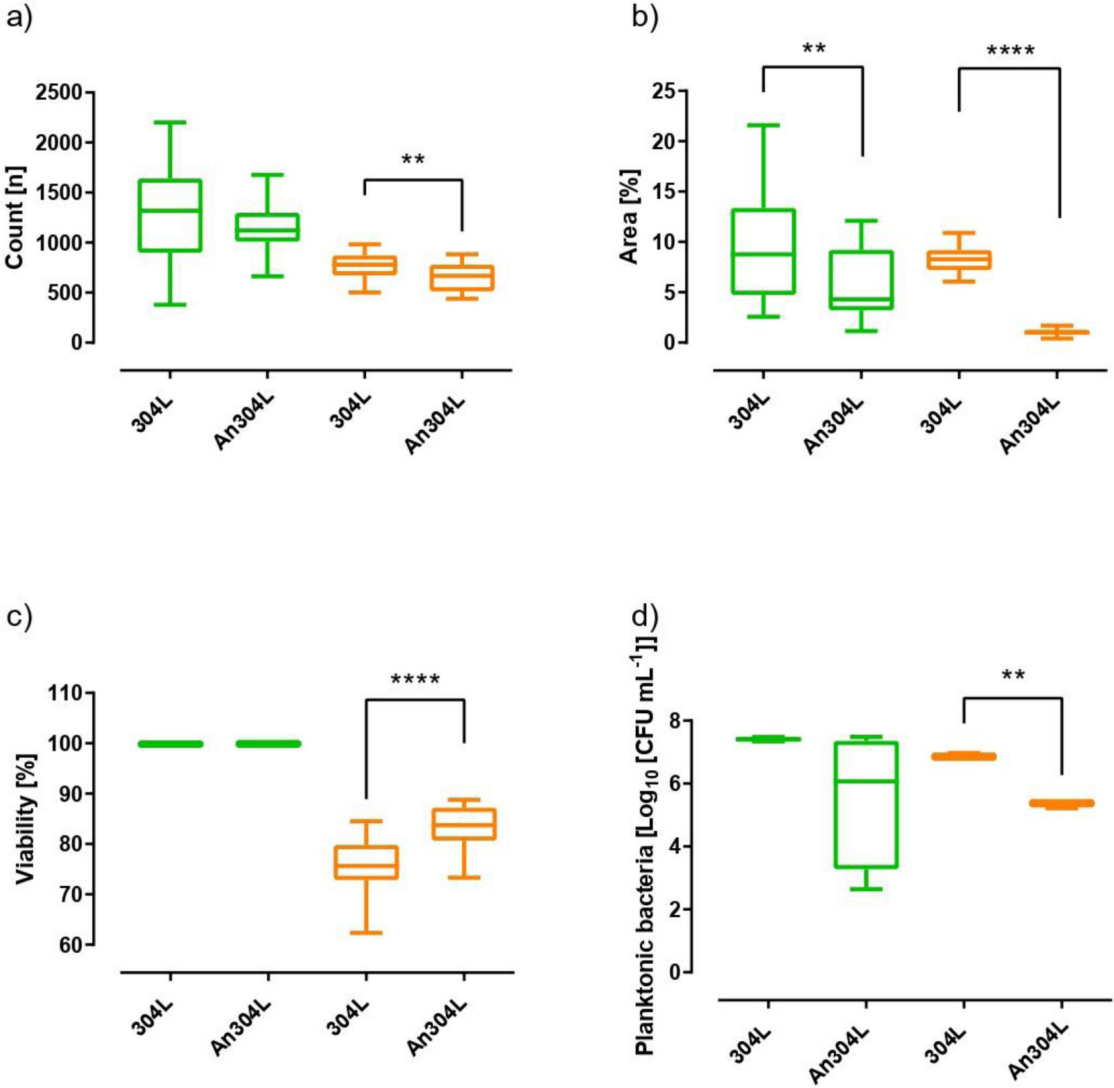
Bacterial counts (**a**), area (b), adhered bacterial viability (**c**), and bacterial concentration in the supernatant (**d**) of *P. aeruginosa* (Pa) (green) and *S. maltophilia* (Sm) (orange) from the non-anodized 304L (304L) and anodized 304L (An304L). *P < 0.05, **P < 0.01, ***P < 0.001, and ****P < 0.0001.

Figure 5 shows the results of the bacterial adherence study. The variables measured in the experiment were count (*n*), area (%), viability (%), and concentration of planktonic bacteria in the supernatant (CFU mL^−1^). A lower adherence on the anodized surface was observed for both *P. aeruginosa* and *S. maltophilia* compared to non-anodized 304L steel, since the area decreased significantly by 50.83% and 87.7%, respectively. Additionally, the count of *S. maltophilia* was significantly reduced by 14%. Colony-forming units per milliliter in the supernatant of *S. maltophilia* were significantly reduced by 1.48log10, while for *P. aeruginosa*, the reduction of 1.35log10 was not statistically significant. These results suggest a potential bactericidal effect of anodized 304L steel when compared to non-anodized 304L steel, even in the case of *P. aeruginosa*. Additionally, the findings suggest that the anodized surface has an anti-adhesive property for these two bacteria.

Interestingly, difference in the viability of both species has been shown (measured by BacLight live/dead stain). There is no clear explanation for this difference. It could be due to the different susceptibility of the two species to the compounds in the anodic layer, the different adaptive responses to environmental changes, or even the fact that the different species may have different behavior when colonizing different materials, as has been the case with other organisms (46).

**Fig 6:**
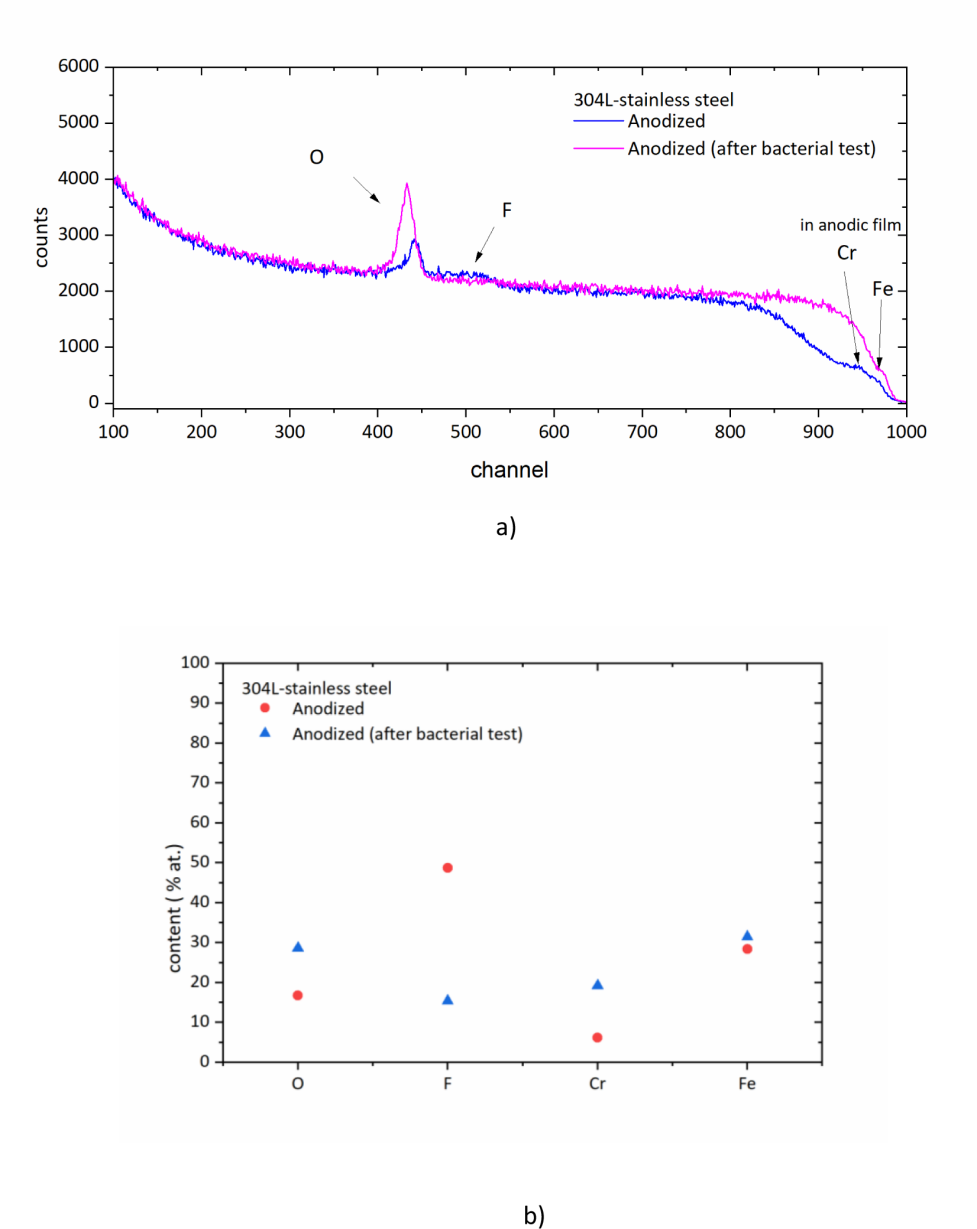
**(a**) Comparison of RBS spectra of anodized 304L stainless-steel RBS before and after bacterial test. (**b**) Variation in composition of the anodic film obtained from RBS simulation.

After the bacterial test, the specimens were analyzed by RBS in order to evaluate whether the compositional changes in the anodic layer may explain the antibacterial properties observed (Fig. 6a). As it can be seen, the spectrum shows a notabe increase in the intensity of the oxygen yield while the fluorine reduces. Following the bacterial tests, the F content in the anodic film decreases from ~49 at.% to ~15 at.%, while the oxygen content increases from ~17 at.% to ~29 at.% (Fig. 6b). According to previous works of the authors performed on Ti alloys fluoride contents ranging from 6 at.% to 12 at.% are enough to provide antibacterial properties to titanium alloy surfaces (25, 38, 47). So, it is expected that the stainless-steel surface still keeps antibacterial properties.

**Fig 7:**
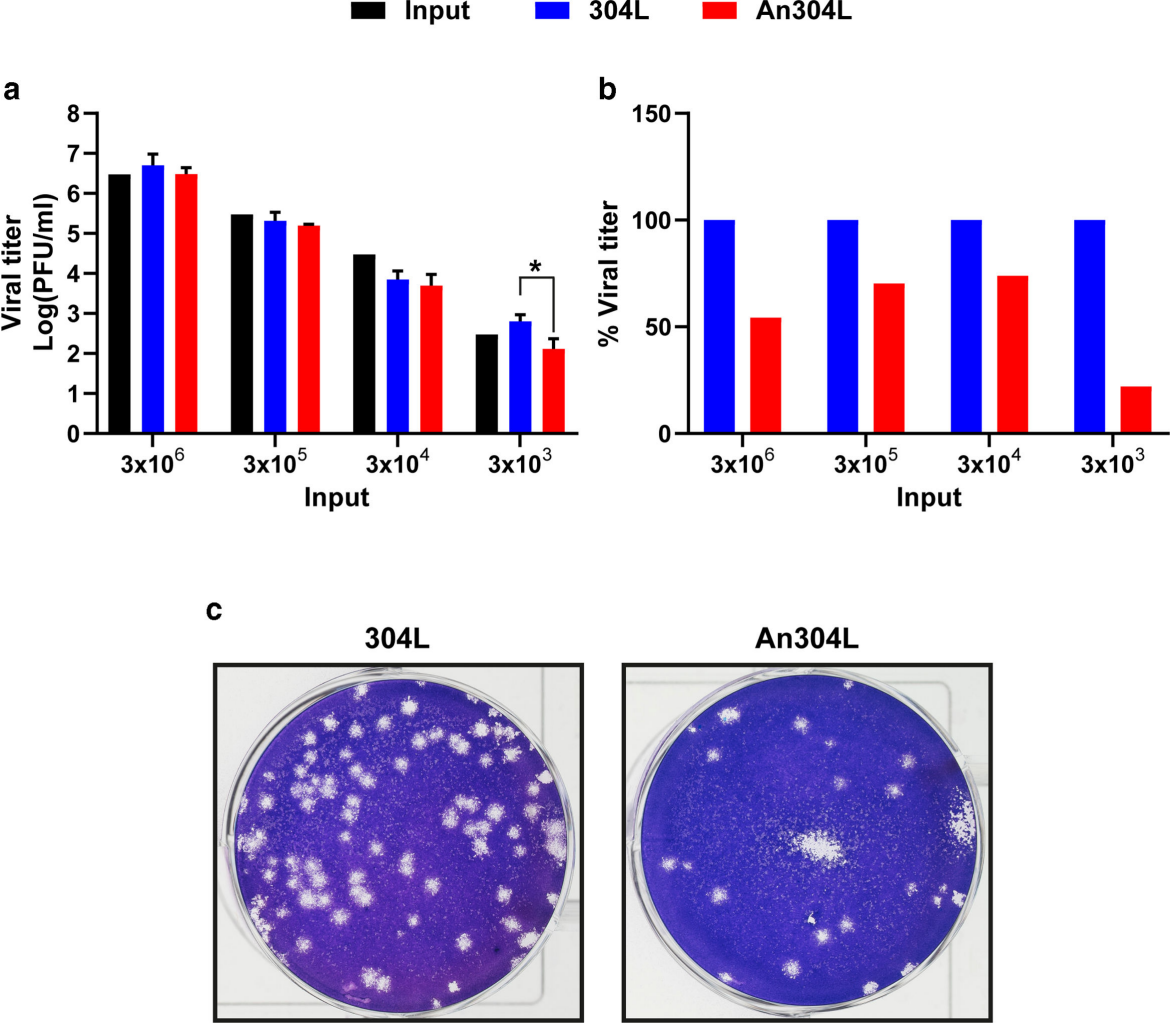
Inactivation of coronavirus HCoV-229E-GFP on metal surfaces. Virus (100 µL) with the total number of infection units indicated in the abscissa (in PFU/mL) was applied on 304L stainless and anodized 304L (An304L) (surface area = 1.77 cm²). After 1 h, samples were eluted and titrated as described in the “Cells and viruses” under Materials and Methods. (**a and b**) Average logarithmic values and standard deviation; the statistical significance was evaluated by applying a *t*-test (*P < 0.05). (**c**) Representative plaque assay that shows the reduction in viral infectivity upon application of a virus sample either to 304L or An304L samples.

Figure 7 showed that incubation in anodized surfaces with F reduces the viral titer of the coronavirus HCoV-229E-GFP. The inactivating effect of F was observed over a 10^3^-fold range of input infectivity, although the maximum decrease appears to occur with a low virus titer, a situation expected for most environmental contamination events. For example, enveloped viruses survive on surfaces with much longer half-lives when they are at higher concentrations (48). Treatment did not alter the plaque size of the surviving virus (Fig. 7b). It is remarkable that the F treatment was effective in inactivating a viral pathogen and two bacterial species. However, the diversity of the microbial and viral worlds begs for studies of inactivation with additional pathogens.

As the anodizing process modifies both the roughness and the F content in the metal surface, both parameters could be responsible for the observed antimicrobial properties. However, surface roughness values of 200 nm were described by Bollenl et al. (49) as the threshold value for roughness to influence on bacterial adhesion. Since the roughness of the anodized 304L stainless steel is about ~101 nm, the antibacterial properties of the anodic film appear to be inherent to the chemical activity of the fluoride. This result is consistent with our previous results on Ti6Al4V (25). Nevertheless, the role of roughness cannot be completely ruled out in the case of virus inactivation, although according to the literature evaluations on the role of the surface nanoscale on virus viability, the inactivation efficiency may depend on the type of virus tested.

### Conclusion

The anodic films grown on 304L stainless steel in an EG solution containing 0.1 M NH_4_F and 0.1 M H_2_O at 50 V, 5°C in static conditions for 15 min show a nanoporous structure with a high fluorine content. The anodic layer is mainly composed of iron fluoride hydroxide, chromium oxide, and chromium fluoride.

The fluoride anodic film on 304L stainless steel exhibits antibacterial properties showed by reduced adherence for both *P. aeruginosa* and *S. maltophilia* compared to non-anodized 304L stainless steel. Moreover, the fluoride anodic film displays a potential bactericidal effect in the case of *S. maltophilia*, evidenced by a significant 1.48log10 reduction in CFU mL^−1^ in the supernatant of anodized samples. Similarly, anodized 304L stainless steel reduces the viral titer of the coronavirus HCoV-229E-GFP, with an inactivation efficiency that is more pronounced when low numbers of infectious units are applied to the metal surface. The lower content of F in the anodic film after the bacterial tests points out fluoride as the antimicrobial agent. Further studies are needed to evaluate the effect of this anodization process on biofilm development by environmental bacteria.
